# Severe Murine Typhus with Pulmonary System Involvement

**DOI:** 10.3201/eid2008.131421

**Published:** 2014-08

**Authors:** Thomas W. van der Vaart, Pieter P.A.M. van Thiel, Nicole P. Juffermans, Michèle van Vugt, Suzanne E. Geerlings, Martin P. Grobusch, Abraham Goorhuis

**Affiliations:** Academic Medical Center, Amsterdam, the Netherlands

**Keywords:** typhus, endemic flea-borne, murine typhus, Rickettsia typhi, acute respiratory distress syndrome, respiratory insufficiency, bacteria

## Abstract

We encountered a case of severe murine typhus complicated by acute respiratory distress syndrome. To determine worldwide prevalence of such cases, we reviewed the literature and found that respiratory symptoms occur in ≈30% of murine typhus patients. In disease-endemic areas, murine typhus should be considered for patients with respiratory symptoms and fever.

Murine typhus (endemic typhus) is a febrile illness caused by fleaborne *Rickettsia typhi*; it occurs mainly in environments where rats and humans live in close proximity. Murine typhus is found worldwide, but most reported cases originate from Southeast Asia, the Mediterranean region, and the United States. Among travelers, murine typhus is most frequently associated with travel to Southeast Asia (*1*). Recently, 2 cases of severe murine typhus with pulmonary manifestations have been reported (*2*,*3*). Near the same time, the Academic Medical Center (Amsterdam, the Netherlands) admitted a patient with severe murine typhus and respiratory failure. On the basis of these 3 cases, we hypothesized that pulmonary system involvement of murine typhus might be more common than previously assumed. We conducted this study because data on prevalence of pulmonary involvement in murine typhus are rarely reported. We therefore describe a clinical case and summarize the published literature on the pulmonary aspects of murine typhus.

## The Study

In February 2012, a previously healthy 40-year-old man visited the Academic Medical Center outpatient department, reporting fever, headache, sweating, and nausea. The signs and symptoms had started 1 day earlier, on the day of his return from a 1-month holiday in Borneo. He reported frequent insect bites and exposure to fresh water. He had taken malaria chemoprophylaxis as recommended, and his vaccinations were up to date. Physical examination indicated that he was afebrile, was hemodynamically stable, and had a discrete macular rash on the trunk but no eschar. Laboratory results showed a hemoglobin concentration (16.8 g/dL) within reference range, a leukocyte count of 4,700 cells/mm^3^ with lymphopenia (1,090 cells/mm^3^), and thrombocytopenia (116,000 cells/mm^3^). C-reactive protein (42 mg/L) and serum creatinine (1.32 mg/dL) concentrations were moderately elevated. A thick smear showed no plasmodia, and a dengue antigen test result was negative. By the next day, the patient’s condition had deteriorated; he was experiencing chills, his temperature was 39°C, and the rash had become more pronounced. He was admitted to the hospital and given doxycycline (200 mg twice a day) for suspected rickettsiosis or leptospirosis. After admission, his condition deteriorated further; increasing dyspnea progressed to respiratory failure, necessitating intubation and admission to the intensive care unit on the second day after admission.

Chest radiographs revealed bilateral interstitial abnormalities (Figure). His condition fit a diagnosis of acute respiratory distress syndrome (ARDS). Empirical treatment was expanded to include broad-spectrum antimicrobial drugs and oseltamivir.

**Figure:**
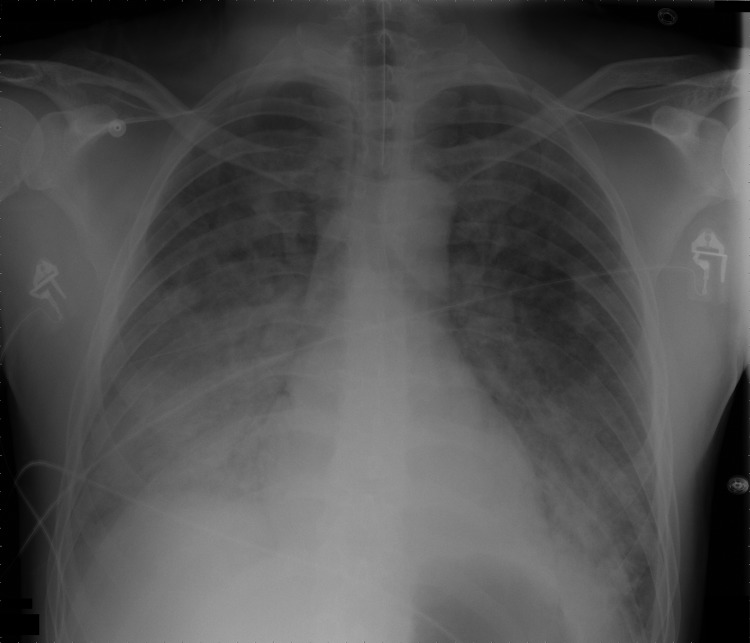
Chest radiograph of 40-year-old man with acute respiratory distress syndrome as a complication of murine typhus.

On the fourth day of intubation, the patient’s condition improved; he was extubated 1 day later. Cultured blood, urine, and bronchial fluid remained sterile, and test results for *Leptospira*, *Legionella*, influenza virus, and HIV were negative. All antimicrobial drugs except doxycycline were discontinued; doxycycline was continued for a total of 14 days.

Serum collected 1 day after admission showed weakly positive IgG against *R. typhi;* after 7 days, the immunofluorescent antibody titer had increased 4-fold (from 1:64 to >1:256). The patient recovered completely and was doing well at his last follow-up visit.

To determine prevalence of such cases, we conducted a search of published studies mentioning pulmonary manifestations of murine typhus (details in the Technical Appendix). From 779 records, we selected 22 cohort studies and 18 case studies that, according to title and abstract, were relevant to our research question. We differentiated between studies with individual patient data (case reports and case series) and studies without individual patient data (cohort studies). For each study, we recorded year of publication, study design, and country of infection. We also recorded the presence of pulmonary involvement, defined as cough and any mention of an abnormal finding on chest radiograph, without further distinction.

An overview of study characteristics detailing prevalence of cough and chest radiograph abnormalities is provided in the Technical Appendix Table 1. Two studies were prospective population-based studies of the causative agent of fever of unknown origin. The remaining 20 studies were all *Rickettsia* spp. specific; in 17 of these studies, patients had been recruited retrospectively from hospital databases or chart reviews.

The 22 study reports that contained data on the presence or absence of cough together accounted for 1,060 patients with murine typhus. The prevalence of cough among these patients ranged from 0 to 66%. Mean prevalence (all patients from all studies combined) of cough was 30.1% (95% CI 23.3–36.9).

Data on presence or absence of radiographic abnormalities were mentioned in 9 study reports (*4*–*12*). Taken together, these studies evaluated 621 patients and 104 chest radiographs showing abnormalities, leading to a prevalence rate of chest radiograph abnormalities of 16.7% (95% CI 8.21–25.5). The cohort studies reported 2 cases of ARDS, 1 with a fatal outcome. The Table shows the full-text descriptions of chest radiograph abnormalities.

**Table:** Pulmonary manifestations of murine typhus reported from cohort studies*

Reference	Year	Region	No. cases	No. chest radiographs	No. chest radiographs showing abnormalities	Details
							(*4*)	1999	Mediterranean	104	NM	8	7 cases of pneumonitis, 1 case of ARDS
(*5*)	2001	USA	97	81	10	Radiographic evidence of pneumonitis in 10/81 cases
(*6*)	2004	Mediterranean	87	NM	6	4 cases of pulmonary infiltrates, 2 cases of pleural effusion
(*7*)	2008	Asia	50	16	6	6 cases of pulmonary infiltrates
(*8*)	2009	Mediterranean	41	NM	22	Abnormal chest radiographs for 22 patients
(*9*)	2009	Asia	28	15	9	9 bilateral reticulonodular infiltrates
(*10*)	2012	Mediterranean	90	NM	15	13 cases of interstitial pneumonia, 2 cases of pleural effusion
(*11*)	2012	Asia	81	49	16	15 cases of pulmonary infiltrates, 1 case of ARDS
(*12*)	2013	Mediterranean	43	39	12	2 cases of alveolar infiltrates, 10 cases of interstitial infiltrates


*NM, not mentioned; ARDS, acute respiratory distress syndrome.

Pulmonary manifestations were also documented by the case studies. Among these studies, 2 patients had ARDS, 7 had bilateral pulmonary infiltrates, 5 had unilateral pulmonary infiltrates, 1 had pulmonary embolism, and 1 had respiratory failure (no chest radiograph was reported) (Technical Appendix Table 2). We also found that cough occurred more commonly among patients in studies conducted in Asia (99 [38.2%] of 259 patients) than among those in studies conducted in the Mediterranean region (118 [25.8%] of 457) and North America (56 [23.3%] of 240).

In the literature, we found 7 reported cases of respiratory distress associated with murine typhus (including the case reported here), 2 of which described respiratory distress not classified as ARDS (*2*,*13*). Of these 7 case-patients with ARDS/respiratory distress, 5 (71%) patients were from Asia (*2*,*3*,*11*,*13*), 1 was from the Mediterranean region (*4*), and 1 was from the United States (*14*).

## Conclusions

Cough and chest radiograph abnormalities were frequent manifestations of murine typhus. For cough associated with murine typhus, we found a prevalence rate of ≈30%. The prevalence of chest radiograph abnormalities was more difficult to ascertain because this result was less often reported and more influenced by bias. The pulmonary aspects of *R. typhi* infection are probably the result of damaged pulmonary microcirculation, leading to pulmonary edema.

Severe pulmonary manifestations of murine typhus are thought to be rare. The case reported here is unusual in that the symptoms progressed rapidly and the response to doxycycline was relatively slow. It is worth noting that we found no more than 2 reported cases of fatal murine typhus associated with pulmonary system disease; both were the result of severe disease complicated by ARDS.

In addition, we noted possible geographic variation in pulmonary manifestations. Most cases of severe murine typhus with pulmonary manifestations originated in Asia, and cough was more frequently a symptom among patients in Asia.

The primary strength of our study is the extensive literature search, which covered studies from different parts of the world and included cohort studies and case series. The main limitation of this study is the retrospective nature of the data collection for most studies, which is prone to bias and renders meaningful statistical analysis of results impossible. Therefore, prospective studies evaluating pulmonary manifestations of murine typhus and possible geographic variation are needed.

Although murine typhus usually follows a benign course, severe disease with pulmonary manifestations, including ARDS, can occur, as described for the patient reported here. We suggest that murine typhus should be included in the differential diagnosis for any patient who has a fever and respiratory signs and who has been in a typhus-endemic area within the incubation period.

Technical Appendix Flowchart of study selection for review, table of cohort studies of murine typhus, and table of case studies detailing chest radiograph abnormalities.
