# Supplementary material for: Severe Murine Typhus with Pulmonary System Involvement
Source: Emerg Infect Dis. 2014 Aug;20(8):1375–7. doi: 10.3201/eid2008.131421 (PMC4111165; doi:10.3201/eid2008.131421)
Supplement: Technical Appendix — Flowchart of study selection for review, table of cohort studies of murine typhus, and table of case studies detailing chest radiograph abnormalities. [file 13-1421-Techapp-s1.pdf]

Article DOI: http://dx.doi.org/10.3201/eid2008.131421

Severe Murine Typhus with Pulmonary System Involvement

Technical Appendix

Search Strategy

We searched MEDLINE with the search terms “Rickettsia typhi”[Mesh] OR (rickettsi*[tiab] AND typhi[tiab]) OR “Typhus, Endemic Flea-Borne”[Mesh] OR (murine[tiab] AND typhus[tiab]) OR (flea-borne[tiab] AND typhus[tiab]) OR endemic typhus[tiab] for articles published from 1 January 1975 to 1 January 2014. Only articles published in English or in Dutch were included. Articles were first selected on the basis of title and abstract, and subsequently on the possibility of containing data on pulmonary symptoms of murine typhus. Full texts of selected articles were read to identify studies containing patient data. Technical Appendix Figure 1 details the selection process.

Technical AppendixTable 1. Overview of cohort studies of murine typhus

Reference	Year	Country	Patient sample	No. cases	Prevalence of cough, %	Prevalence of chest radiograph abnormalities , %
(*1*)	1982	Kuwait	Retrospective cohort	104	44	
(*2*)	1991	USA	Retrospective cohort	80	35	
(*3*)	1991	Thailand	Retrospective cohort	10	40	
(*4*)	1992	Greece	Retrospective cohort	49	37	
(*5*)	1999	Spain	Retrospective cohort	104	25	7
(*6*)	2000	USA	Retrospective cohort	30	40	
(*7*)	2001	Singapore	Retrospective cohort	21	43	
(*8*)	2001	USA	Retrospective cohort	97	10	10
(*9*)	2003	Sri Lanka	Prospective cohort	2	0	
(*10*)	2003	Thailand	Prospective cohort	4	25	
(*11*)	2004	Greece	Retrospective cohort	87	30	7
(*12*)	2004	Greece	Retrospective cohort	22	27	
(*13*)	2006	Laos	Prospective cohort	41	34	
(*14*)	2007	Greece	Retrospective cohort	21	19	
(*15*)	2008	Nepal	Retrospective cohort	50	66	12
(*16*)	2009	Greece	Retrospective cohort	41	10	54
(*17*)	2009	Thailand	Retrospective cohort	28	32	32
(*18*)	2010	USA	Retrospective	33	18	

Reference	Year	Country	Patient sample	No. cases	Prevalence of cough, %	Prevalence of chest radiograph abnormalities , %
			cohort			
(*19*)	2012	Greece	Prospective cohort	90	32	14
(*20*)	2012	Taiwan	Retrospective cohort	81	26	19
(*21*)	2012	Nepal	Prospective cohort	22	36	
(*22*)	2013	Tunisia	Retrospective cohort	43	12	28

Technical Appendix Table 2. Overview of case studies detailing chest radiograph abnormalities*

Reference	Year	Region	No. patients	Details as provided in full text
(*23*)	1986	Mediterranean	1	Pulmonary embolism
(*24*)	1989	USA	1	ARDS
(*25*)	1997	Asia	1	Unilateral pulmonary infiltrates
(*26*)	2003	USA	1	Bilateral pulmonary edema and pleural effusion
(*27*)	2005	Mediterranean	4	3 cases of interstitial pneumonia, 1 case of alveolar infiltrates
(*28*)	2010	Asia	1	Unilateral pulmonary infiltrates
(*29*)	2010	USA	1	Unilateral pulmonary infiltrates
(*30*)	2011	Asia	1	Bilateral pulmonary infiltrates
(*31*)	2011	Asia	1	Bilateral pulmonary infiltrates
(*32*)	2011	Asia	1	Respiratory failure without a reported chest radiograph
(*33*)	2012	Mediterranean	2	unilateral pulmonary infiltrates
(*34*)	2013	Asia	1	ARDS

*Six more articles only mention cough as a symptom and are not included in this table. References are available on request. ARDS, acute respiratory distress syndrome.

References

1. Al-Awadi AR, Al-Kazemi N, Ezzat G, Saah AJ, Shepard C, Zaghloul T, et al. Murine typhus in Kuwait in 1978. Bull World Health Organ. 1982;60:283–9. PubMed

2. Dumler JS, Taylor JP, Walker DH. Clinical and laboratory features of murine typhus in south Texas, 1980 through 1987. JAMA. 1991;266:1365–70. PubMed http://dx.doi.org/10.1001/jama.1991.03470100057033

3. Silpapojakul K, Chupuppakarn S, Yuthasompob S, Varachit B, Chaipak D, Borkerd T. Scrub and murine typhus in children with obscure fever in the tropics. Pediatr Infect Dis J. 1991;10:200–3. PubMed http://dx.doi.org/10.1097/00006454-199103000-00006

4. Tselentis Y, Babalis TL, Chrysanthis D, Gikas A, Chaliotis G, Raoult D. Clinicoepidemiological study of murine typhus on the Greek island of Evia. Eur J Epidemiol. 1992;8:268–72. PubMed http://dx.doi.org/10.1007/BF00144812

5. Bernabeu-Wittel M, Pachon J, Alarcon A, Lopez-Cortes LF, Viciana P, Jimenez-Mejias ME, et al. Murine typhus as a common cause of fever of intermediate duration: a 17-year study in the south of Spain. Arch Intern Med. 1999;159:872–6. PubMed http://dx.doi.org/10.1001/archinte.159.8.872

6. Fergie JE, Purcell K, Wanat D. Murine typhus in South Texas children. Pediatr Infect Dis J. 2000;19:535–8. PubMed http://dx.doi.org/10.1097/00006454-200006000-00009

7. Ong AK, Tambyah PA, Ooi S, Kumarasinghe G, Chow C. Endemic typhus in Singapore–a re-emerging infectious disease? Singapore Med J. 2001;42:549–52. PubMed

8. Whiteford SF, Taylor JP, Dumler JS. Clinical, laboratory, and epidemiologic features of murine typhus in 97 Texas children. Arch Pediatr Adolesc Med. 2001;155:396–400. PubMed http://dx.doi.org/10.1001/archpedi.155.3.396

9. Kularatne SA, Edirisingha JS, Gawarammana IB, Urakami H, Chenchittikul M, Kaiho I. Emerging rickettsial infections in Sri Lanka: the pattern in the hilly Central Province. Trop Med Int Health. 2003;8:803–11. PubMed http://dx.doi.org/10.1046/j.1365-3156.2003.01108.x

10. Parola P, Miller RS, McDaniel P, Telford SR III, Rolain JM, Wongsrichanalai C, et al. Emerging rickettsioses of the Thai-Myanmar border. Emerg Infect Dis. 2003;9:592–5. PubMed http://dx.doi.org/10.3201/eid0905.020511

11. Gikas A, Doukakis S, Pediaditis J, Kastanakis S, Manios A, Tselentis Y. Comparison of the effectiveness of five different antibiotic regimens on infection with Rickettsia typhi: therapeutic data from 87 cases. Am J Trop Med Hyg. 2004;70:576–9. PubMed

12. Hernández-Cabrera M, Angel-Moreno A, Santana E, Bolanos M, Frances A, Martin-Sanchez MS, et al. Murine typhus with renal involvement in Canary Islands, Spain. Emerg Infect Dis. 2004;10:740–3. PubMed http://dx.doi.org/10.3201/eid1004.030532

13. Phongmany S, Rolain JM, Phetsouvanh R, Blacksell SD, Soukkhaseum V, Rasachack B, et al. Rickettsial infections and fever, Vientiane, Laos. Emerg Infect Dis. 2006;12:256–62. PubMed http://dx.doi.org/10.3201/eid1202.050900

14. Koliou M, Psaroulaki A, Georgiou C, Ioannou I, Tselentis Y, Gikas A. Murine typhus in Cyprus: 21 paediatric cases. Eur J Clin MicrobiolIinfectDis. 2007; ;26;491–3. PMID: 17554571

15. Zimmerman MD, Murdoch DR, Rozmajzl PJ, Basnyat B, Woods CW, Richards AL, et al. Murine typhus and febrile illness, Nepal. Emerg Infect Dis. 2008;14:1656–9. PubMed http://dx.doi.org/10.3201/eid1410.080236

16. Gikas A, Kokkini S, Tsioutis C, Athenessopoulos D, Balomenaki E, Blasak S, et al. Murine typhus in children: clinical and laboratory features from 41 cases in Crete, Greece. ClinMicrobiol Infect.2009;15 (Suppl 2):211–2. PMID19374652

17. Suputtamongkol Y, Suttinont C, Niwatayakul K, Hoontrakul S, Limpaiboon R, Chierakul W, et al. Epidemiology and clinical aspects of rickettsioses in Thailand. Ann N Y Acad Sci. 2009;1166:172–9. PubMed http://dx.doi.org/10.1111/j.1749-6632.2009.04514.x

18. Adjemian J, Parks S, McElroy K, Campbell J, Eremeeva ME, Nicholson WL, et al. Murine typhus in Austin, Texas, USA, 2008. Emerg Infect Dis. 2010;16:412–7. PubMed http://dx.doi.org/10.3201/eid1603.091028

19. Chaliotis G, Kritsotakis EI, Psaroulaki A, Tselentis Y, Gikas A. Murine typhus in central Greece: epidemiological, clinical, laboratory, and therapeutic-response features of 90 cases. Int J Infect Dis. 2012;16:e591–6. **PMID: 22658872 doi: 10.1016/j.ijid.2012.03.010.**

20. Chang K, Chen YH, Lee NY, Lee HC, Lin CY, Tsai JJ, et al. Murine typhus in southern Taiwan during 1992–2009. Am J Trop Med Hyg. 2012;87:141–7. PubMed http://dx.doi.org/10.4269/ajtmh.2012.11-0465

21. Pradhan R, Shrestha U, Gautam SC, Thorson S, Shrestha K, Yadav BK, et al. Bloodstream infection among children presenting to a general hospital outpatient clinic in urban Nepal. PLoS ONE. 2012;7:e47531. PubMed http://dx.doi.org/10.1371/journal.pone.0047531

22. Znazen A, Hammami B, Mustapha AB, Chaari S, Lahiani D, Maaloul I, et al. Murine typhus in Tunisia: a neglected cause of fever as a single symptom. Med Mal Infect. 2013;43:226–9. PubMed http://dx.doi.org/10.1016/j.medmal.2013.02.007

23. Potasman I, Bassan HM. Pulmonary embolism complicating murine typhus. J R Soc Med. 1986;79:367–8. PubMed

24. Walker DH, Parks FM, Betz TG, Taylor JP, Muehlberger JW. Histopathology and immunohistologic demonstration of the distribution of *Rickettsia typhi* in fatal murine typhus. Am J Clin Pathol. 1989;91:720–4. PubMed

25. Lu TM, Kuo BI, Chung YM, Liu CY. Murine typhus presenting with multiple white dots in the retina. Scand J Infect Dis. 1997;29:632–3. PubMed http://dx.doi.org/10.3109/00365549709035910

26. Centers for Disease Control and Prevention. Murine typhus—Hawaii, 2002. MMWR Morb Mortal Wkly Rep. 2003;52:1224–6. PubMed

27. Letaief AO, Kaabia N, Chakroun M, Khalifa M, Bouzouaia N, Jemni L. Clinical and laboratory features of murine typhus in central Tunisia: a report of seven cases. Int J Iinfect Dis. 20059:331-4. **PMID: 16054415**

28. Lin SY, Wang YL, Lin HF, Chen TC, Chen YH, Lu PL. Reversible hearing impairment: delayed complication of murine typhus or adverse reaction to azithromycin? J Med Microbiol. 2010;59:602–6. PubMed http://dx.doi.org/10.1099/jmm.0.013813-0

29. Restrepo MI, Vasquez EM, Echeverri C, Fiebelkorn KR, Anstead GM. Fibrin ring granulomas in Rickettsia typhi infection. Diagn Microbiol Infect Dis. 2010;66:322–5. PubMed http://dx.doi.org/10.1016/j.diagmicrobio.2009.10.019

30. Schulze MH, Keller C, Muller A, Ziegler U, Langen HJ, Hegasy G, et al. *Rickettsia typhi* infection with interstitial pneumonia in a traveler treated with moxifloxacin. J Clin Microbiol. 2011;49:741–3. PubMed http://dx.doi.org/10.1128/JCM.01201-10

31. Stockdale AJ, Weekes MP, Kiely B, Lever AM. Case report: Severe typhus group rickettsiosis complicated by pulmonary edema in a returning traveler from Indonesia. Am J Trop Med Hyg. 2011;85:1121–3. PubMed http://dx.doi.org/10.4269/ajtmh.2011.11-0340

32. Tan L, Beersma TM, van Beek Y, van Genderen PJ. [Two travellers suffering from typhus]. Ned Tijdschr Geneeskd. 2011;155:A3845. PubMed

33. Velasco-Tirado V, Hernandez-Cabrera M, Pisos-Alamo E, Perez-Arellano JL. Rickettsia typhi. A new causative agent of round pneumonia in adults. Enferm Infecc Microbiol Clin. 2012;30:427–8. PubMed http://dx.doi.org/10.1016/j.eimc.2012.02.008

34. Sakamoto N, Nakamura-Uchiyama F, Kobayashi K, Takasaki T, Ogasawara Y, Ando S, et al. Severe murine typhus with shock and acute respiratory failure in a Japanese traveler after returning from Thailand. J Travel Med. 2013;20:50–3. PubMed http://dx.doi.org/10.1111/j.1708-8305.2012.00678.x

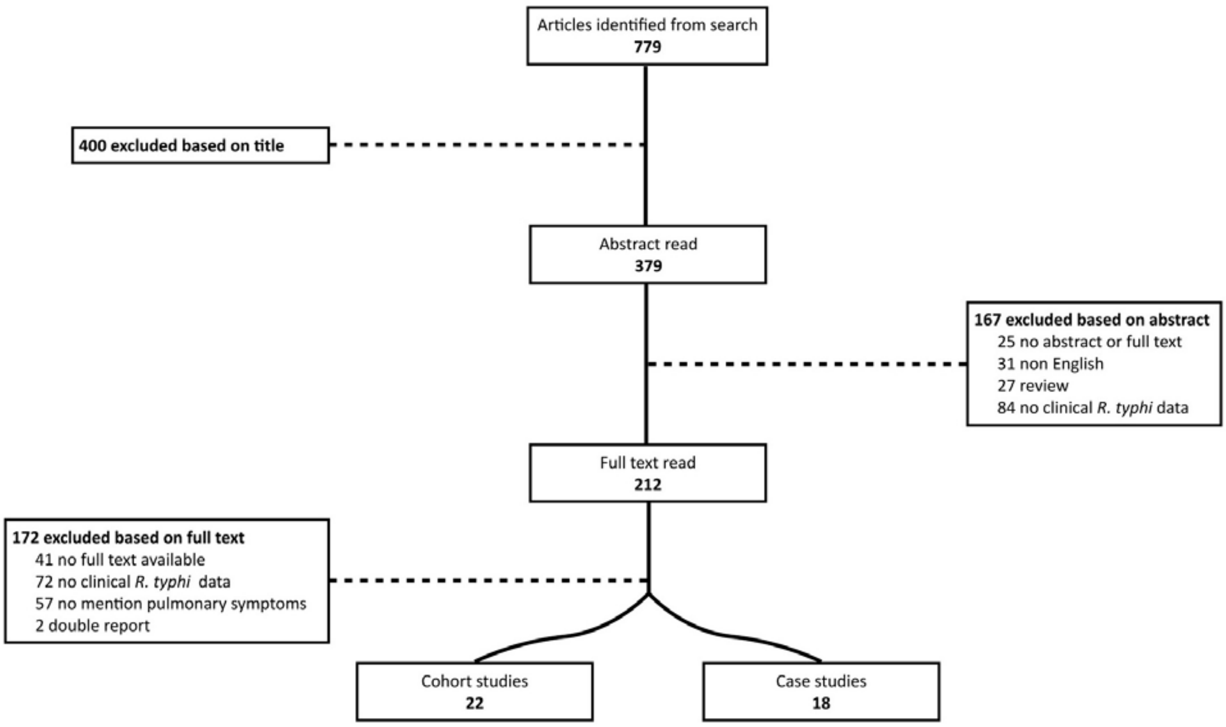

Technical Appendix Figure. Flowchart of study selection process.
